# Significance of preadmission quality of life for mortality in the ICU: a prospective cohort study

**DOI:** 10.1186/cc12419

**Published:** 2013-03-19

**Authors:** RB Bukan, A Moeller, M Henning, K Mortensen, T Klausen, T Waldau

**Affiliations:** 1 Herlev University Hospital, Herlev, Denmark; 2 University of Copenhagen, Denmark

## Introduction

Assessing whether a critically ill patient should be admitted to an ICU remains difficult and mortality amongst ICU patients is high. To render intensive care with no prospect of success is an immense emotional burden for both patient and relatives, and a great socioeconomic burden for society as well. Therefore, validated strategies that can help identify patients who will benefit from intensive care are in demand. This study seeks to investigate whether preadmission quality of life can act as a predictor of mortality amongst patients admitted to the ICU.

## Methods

All patients (>18 years) admitted to the ICU for more than 24 hours are included. In order to assess preadmission quality of life, the patient or close relatives complete the Short-Form 36 (SF-36) within 72 hours after ICU admission. Mortality is evaluated from ICU admission until 30 days hereafter. Logistic regression and receiver operating characteristic analyses are employed to assess predictive value for mortality using five models: SF-36 Physical Component Summary (PCS) and APACHE II (model A), SF-36 PCS (model B), SF-36 General Health (GH) and APACHE II (model C), SF-36 GH (model D), and APACHE II (model E). Classification tables are composed in order to assess sensitivity, specificity, positive and negative predictive values and likelihood ratios.

## Results

Preliminary results, based upon data from 175 included patients, reveal an ICU mortality of 12.6% and 30-day mortality of 22.9%. No patients were lost to follow-up. When the Physical Component of SF-36 (PCS) was used as an estimate of preadmission quality of life, the area under the curve for model B (AUC = 0.80) was comparable with model E (AUC = 0.81), and better than model A (AUC = 0.85). The General Health item of SF-36 (GH), used as an estimate of preadmission quality of life, gave an AUC = 0.76 (model D). All models were controlled for sex and age.

## Conclusion

Preliminary results indicate that the SF-36 GH and the SF-36 PCS are as good as APACHE II to predict mortality 30 days after ICU admission. However, in order to conclude whether preadmission quality of life can contribute to triage, by successfully identifying patients suitable for intensive care, final analyses, due in 2013, are awaited. These results will clarify whether future randomized controlled trials, in which preadmission quality of life acts as a supplement to triage, are justifiable.

